# Flow Cytometric Sorting of Infected Erythrocytes Demonstrates Reliable Detection of Individual Ring-Stage *Plasmodium falciparum* Parasites by *Plasmodium* 18S rRNA Reverse Transcription Polymerase Chain Reaction

**DOI:** 10.4269/ajtmh.21-1226

**Published:** 2022-04-11

**Authors:** Jokichi Matsubara, Ming Chang, Annette M. Seilie, Sean C. Murphy

**Affiliations:** ^1^ Department of Laboratory Medicine and Pathology, University of Washington, Seattle, Washington;; ^2^ Center for Emerging and Re-emerging Infectious Diseases, University of Washington, Seattle, Washington;; ^3^ Department of Microbiology, University of Washington, Seattle, Washington

## Abstract

Molecular diagnostic tests for *Plasmodium falciparum* parasites are increasingly used to enable ultrasensitive detection of infection in clinical trials and field surveillance studies. Ribonucleic acid (RNA)-based assays targeting 18S rRNA are particularly sensitive with limits of detection reported to comprise a single infected red blood cell (RBC) in a relatively large volume of blood. However, the validation testing at such limiting concentrations is hampered by the so-called Poisson distribution of such rare events, which can lead laboratorians to inaccurately set the limit of detection higher (i.e., less sensitive) than the assay can actually detect. Here we set out to formally demonstrate the analytical sensitivity of the *Plasmodium* 18S rRNA quantitative reverse transcription PCR (qRT-PCR). Fluorescence-activated cell sorting (FACS) was used on synchronous *P. falciparum* cultures doubly stained for DNA and RNA and was followed by qRT-PCR on the individual sorted cells spiked with negative whole blood. Over 95% of individual single-ring infected RBCs were detected by qRT-PCR. The formally measured median 18S rRNA content per individual ring-stage *P. falciparum* parasite was 9,550 copies (interquartile range 8,130–12,300). Thus, one can confidently rely on *Plasmodium* 18S rRNA qRT-PCR to detect one parasite per 50-µL blood sample.

## INTRODUCTION

The *Plasmodium* 18S ribosomal RNA (rRNA) is a biomarker for identifying and quantifying *Plasmodium* sp. in infected humans, mosquito vectors, and animal models.^1^^–^^4^ Ribosomal RNA molecules have been long recognized to be highly expressed in the parasite compared with the DNA gene copies that encode rRNA (rDNA).^5^ Accordingly, a real-time quantitative reverse transcription PCR assay based on this biomarker was first developed in 2012 by the University of Washington (herein called UW qRT-PCR), and the biomarker was recently qualified for use in non-endemic controlled human malaria infection (CHMI) studies by the Food and Drug Administration (FDA).^1^^,^^6^ The UW qRT-PCR assay targets *P. falciparum* parasites in infected blood samples. In contrast to *P. falciparum* trophozoites and schizonts, ring-stage parasites as well as less common gametocytes are not sequestered from circulating blood, and are therefore able to be identified in blood smears and by molecular assays.^7^ Each *P. falciparum* genome contains five 18S rRNA genes (two sexual, two asexual, one pseudogene) and the asexually expressed 18S rRNAs are estimated to be present ∼3 logs higher than rDNA in each ring-stage parasite.^1^^,^^6^^,^^8^^,^^9^

The standard sample for this qRT-PCR assay is 0.05 mL of ethylenediaminetetraacetic acid (EDTA) whole blood or a 0.05 mL dried blood spot. Such samples are lysed in 2 mL concentrated guanidinium-based solution (i.e., NucliSENS lysis buffer, bioMérieux) resulting in the breakdown of any present intact parasites, dissociation of nucleic acid from macromolecular structure within cells, and stabilization of RNA. Prior to this study, the analytical sensitivity of the qRT-PCR assay had been evaluated using serial dilutions of known-parasitemia *P. falciparum* cultures down to approximately one parasite per 0.05 mL sample (i.e., equivalent to 20 parasites/mL). In addition, the sensitivity of the assay appears to reliably detect to even lower concentrations that constitute < 1 parasite per sample when testing was done using thousands of bacteriophage-like Armored RNAs^®^ containing the entire *P. falciparum* asexual 18S rRNA sequence (Pf-Armored RNA).^6^ To push the sensitivity further, a previous study using this approach also showed that a single parasite was likely detectable in even larger blood sample volumes (e.g., 0.5 mL).^8^

**Figure 1.**
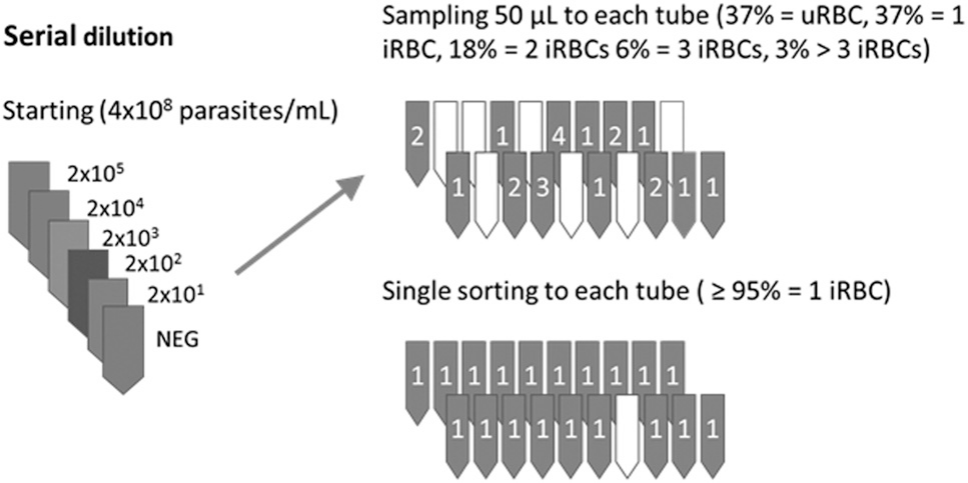
Theoretical outcome of repeated sampling to obtain single infected red blood cells (iRBCs) by standard pipetting vs. fluorescence-activated cell sorting (FACS) for individual iRBCs. Parasite standard curves are usually prepared by repeated dilution into uninfected whole blood (left). When diluted as shown, the final dilution at 20 parasites/mL blood corresponds to an average of one infected RBC (iRBC) per 50-µL of sampled whole blood. However, actual distribution is best described by Poisson distribution such that some volumetrically pipetted samples will contain zero iRBCs (37% of the time), some will contain a single iRBC as intended (37% of the time), and the remainder will contain two or more iRBCs as shown in the upper right. Fluorescence-activated cell sorting-based preparation of iRBC-containing samples using DNA and RNA staining (lower right) can overcome this sampling bias to prepare a reliable panel of samples at the theoretical limit of detection.

Despite a wealth of data based on hand-diluted standard curves of parasite-containing samples, the possibility remained that fractionated parasites or extracellular parasite 18S rRNAs were contributing to the ultrasensitive performance. In addition, the production of high-quality parasite standard curves were dependent on having extremely synchronous parasite cultures, and even highly synchronous cultures nonetheless result in variable standard curves because of problems of limiting sample statistics. Here, the Poisson distribution describes the likelihood that a certain number of infected red blood cells (herein named iRBC) will be present in a specific sample volume when prepared from a reliably quantified stock. In most molecular assays, a claim of single organism detection implies single copy detection, but that is not the case for *P. falciparum* 18S rRNA qRT-PCR since each parasitized RBC contains thousands of copies of mature 18S rRNA. Despite the abundant target within individual infected cells, sampling bias can affect the perceived analytical detection. At a nominal (average rate of occurrence) concentration of one iRBC per sample, the Poisson distribution predicts that 36.8% of 100 such samples would actually contain zero iRBCs, 36.8% would contain one iRBC, 18.4% would contain two iRBCs, 6.1% would contain three, and so on (Figure 1). This distribution would skew to the right by several factors including inaccurately quantified higher-density samples, and the presence of multiply infected rings as is common in *P. falciparum* culture.

Here, we sought to overcome the problem of sample bias due to pipetting using fluorescence-activated cell sorting (FACS) method to directly demonstrate reliable and precise detection of single *P. falciparum*-infected RBCs using the qRT-PCR assay. The data support the ultrasensitive nature of the assay and indicate that sample volume is the major driver of analytical sensitivity for this assay.

## MATERIALS AND METHODS

### *Plasmodium falciparum* in vitro culture.

The *P. falciparum* 3D7 strain was cultured in type O human erythrocytes using a modified Trager and Jensen method.^10^ RPMI 1640 containing 2 mM L-glutamine and 25 mM HEPES was supplemented with 0.5% Albumax II (GIBCO, San Diego, CA), 26.8 mM NaHCO_3_, 11 mM dextrose, 360 μM hypoxanthine, 5% human type AB sera, and 11 μg/L gentamicin. Cultures were placed in a chamber gassed with 5% O_2_, and 5% CO_2_ (in N_2_) and incubated at 37°C. To prepare synchronous cultures, late-stage trophozoites and schizonts were enriched using a gradient of 65% Percoll^®^ (Sigma-Aldrich, St. Louis, MO) supplemented with RPMI 1640 (Invitrogen) and 5% NaHCO_3_. Resulting mature parasites were resuspended in growth medium to invade fresh erythrocytes at 1–5% hematocrit. After 12 hours, the newly invaded ring-stage parasites were further enriched with 5% D-sorbitol (Sigma-Aldrich) for 15 minutes to eliminate any remaining schizonts. Cultures were washed twice in serum-free media and resuspended in growth medium. Giemsa-stained thin blood smears were used to assess parasitemia and synchrony.

### Deoxyribonucleic acid and RNA staining of live *P. falciparum* cultures.

*Plasmodium falciparum* cultures were centrifuged at 600 × g for 5 minutes and washed twice with MACS buffer (PBS, 2 mM EDTA, 0.5% fetal bovine serum). Washed RBCs were resuspended in MACS buffer containing 3.4 μM Hoechst 33342 (HO) (Invitrogen, Carlsbad, CA) to stain DNA and/or 1 ng/mL thiazole orange (TO) (Polysciences, Warrington, PA) to stain RNA. One aliquot of washed RBCs remained unstained. All samples (HO-stained, TO-stained, dual HO/TO-stained, and unstained samples) were incubated for 30 minutes at 37°C in dark, and then washed twice with MACS buffer before a final resuspension in MACS buffer.

### Imaging flow cytometry of *P. falciparum*-infected cultures.

Imaging flow cytometry (IFC) was performed as described with minor modifications. Live-stained synchronous *P. falciparum* cultures stained by HO and TO were evaluated by IFC using an Imagestream X (Amnis^®^ ImageStream^®^ X Mark II).^11^ HO was excited by a 405 nm laser and detected by a 457/45 filter (DAPI filter). Thiazole orange was excited by a 488 nm blue laser and detected by a 533/55 filter (FITC filter). Gating was performed to identify uninfected RBCs (uRBCs), iRBCs containing one ring-stage parasite (herein named single-ring iRBC), and more mature or multiply infected cells using two-dimensional dot plots. Bright-field, DAPI-, and FITC-filtered IFC images and composite images were generated for each iRBC.

### Fluorescence-activated cell sorting of *P. falciparum*-infected cultures.

Fluorescence-activated cell sorting was performed on a BD FACS Aria II Cell Sorter (Becton Dickinson, Franklin Lakes, NJ), and data were analyzed using FACS DiVa software. Forward scatter (FSC) and side scatter (SSC) were used to identify human RBCs. HO was excited by a 407 nm violet laser and detected by a 440/40 filter. Thiazole orange was excited by a 488 nm blue laser and detected by a 530/30 filter. Infected cultures stained with TO only or HO only served as compensation controls. Infected RBCs containing single ring-stage parasites were gated and sorted from synchronous *P. falciparum* cultures.

Uninfected RBCs and single-ring iRBCs were individually collected into 100 µL of NucliSENS Lysis Buffer in 96 well plates and then stored at −80°C. Thawed samples were diluted in 1.9 mL of lysed whole human blood—a mixture of EDTA-anticoagulated human whole blood and NucliSENS Lysis Buffer at a volumetric ratio of 1:40 for nucleic acid extraction using the Abbott m2000sp instrument as described below.

### *Plasmodium* 18S rRNA qRT-PCR assay.

The published third-generation University of Washington qRT-PCR was performed using the Abbott m2000 platform for nucleic acid extraction and amplification.^6^ Briefly, 50 µL of EDTA-anticoagulated human whole blood was lysed in 2 mL of lysis buffer. One milliliter of this blood lysate was extracted for nucleic acid and targets were amplified by a triplex qRT-PCR targeting *P. falciparum* 18S rRNA (*P. falciparum* qRT-PCR), pan-*Plasmodium* 18S rRNA (pan-*Plasmodium* qRT-PCR), and the human TATA-binding protein (TBP) mRNA (TBP RT-PCR) as an endogenous control. Some experiments also used slight variation on the core assay where the human TBP reagents were omitted (called Gen3.0-NO_TBP) or where an updated generation of the full triplex qRT-PCR was used (Gen3.5) as previously described.^2^ High and low positive controls containing 400,000 and 1,000 cultured *P. falciparum* parasites, respectively and one negative control were included in each extraction/amplification run. Results were monitored by Levey-Jennings (bias) plots over time. Quantification was provided for all assays using a four-level standard curve made of Pf-Armored RNA (Asuragen); results were calculated as log_10_ copies 18S rRNA per mL blood of sample and were converted to estimate parasites/mL blood using our published approach.^1^^,^^6^^,^^9^

## RESULTS

### Visualization of infected RBCs using IFC.

**Figure 2.**
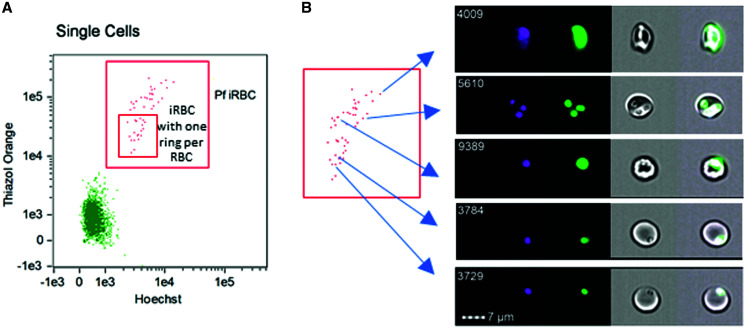
Identification of *Plasmodium falciparum* infected RBCs (iRBCs) by imaging flow cytometry. (**A**) Gating of iRBCs and sub-gate denoting iRBCs containing one ring stage parasite per RBC. (**B**) Images of each iRBC for the specific spot in the two-dimensional dot plot. Hoechst 33342 and thiazole orange dyes were used to stain DNA and RNA, respectively, and fluorescence was emitted with DAPI and FITC filters, respectively. Examples of iRBCs containing a single-ring, multiple rings, a trophozoite or a schizont. Four images are displayed for each iRBC: DAPI-filtered (first column, in pink), FITC-filtered (second column, in green), brightfield (third column), and composite (fourth column) images.

*Plasmodium falciparum* cultures usually contain a mixed population of iRBCs consisting of single and multiple rings, trophozoites, and schizonts (Supplemental Figure 1). Lower frequencies of trophozoites and schizonts were seen in synchronized ring cultures. To sort and visualize iRBC from synchronized cultures, the culture was stained with HO and TO to mark DNA-enriched RBC nuclei and rRNA-enriched cytoplasm, respectively. Both are indicative of iRBCs. This stained material was then assessed by IFC as described.^11^ Infected red blood cells containing single ring stage parasites or more mature stages were gated on DNA and RNA content and sorted from uRBCs (Figure 2A). Imaging flow cytometry bright-field images of what were expected to be single-ring iRBCs clearly showed one individual ring stage parasite per iRBC. Fluorescence images showed co-localization of HO (DNA) and TO (RNA) within each ring (Figure 2B, for example cells #3729 and #3784 as shown). The intensity and area of the HO- and TO-stained fluorescence in such ring stage iRBCs were lower than in more mature or multiply infected iRBCs (Figure 2B).

Bright-field IFC images also showed visible differences between singly versus multiply infected iRBCs. For example, cell #5610 (Figure 2B) shows a representative iRBC containing three rings with three distinct HO- and TO-stained foci inside of each of the three rings. Infected red blood cells containing larger trophozoite/schizont parasites showed even stronger HO- and TO-stained fluorescence and bright field microscopy showed the dense texture of such iRBC (Figure 2B, e.g., Cell 9389 and Cell 4009, respectively). Thus, IFC confirmed that HO- and TO-staining reliably separated individual ring stage parasites from other stages.

### Fluorescence-activated cell sorting isolation and qRT-PCR quantification of 18S rRNA from individual ring stage parasites.

Flow cytometry of synchronous (Supplemental Figure 2A) and asynchronous (Supplemental Figure 2B) cultures showed the as-expected predominance of ring-stage parasite or a broad continuum of all iRBC stages, respectively. For FACS of HO-/TO-stained cells, a small ring stage-only gate was applied to synchronous cultures (Supplemental Figure 2A). Forty uRBCs and 30 single-ring iRBCs were individually sorted into a defined volume of lysis buffer. Each aliquot was combined with uninfected lysed blood, extracted, and tested in multiple qRT-PCR runs. The FACS method was apparently not prone to molecular cross-contamination since all 40 individually sorted uRBCs were negative for both pan-*Plasmodium* and *P. falciparum* 18S rRNA targets in the Gen3.0 qRT-PCR. Among infected RBCs, 29 of 30 (97%) single-ring iRBCs tested positive for both targets. Another 10 uninfected RBCs tested negative and 47 of 48 (98%) single-ring iRBCs tested positive in two runs by the Gen3.5 qRT-PCR. Thus, the positive agreement between sorted single ring stage iRBCs and qRT-PCR positivity for *P. falciparum* 18S rRNA was > 95% across both assays. Further, the specificity of the 18S rRNA qRT-PCRs on sorted uRBCs was 100% for both assays (Table 1).

**Table 1:** Agreement between the FACS and 18S rRNA qRT-PCR assays

		UW Gen3.0 assay		UW Gen3.5 assay	
		Positive	Negative	Positive	Negative
FACS gate	Single ring iRBC	29	1	47	1
	Uninfected RBC	0	40	0	10

FACS = fluorescence-activated cell sorting; iRBC = infected red blood cells.

### Range of 18S rRNA content in ring stage parasites.

**Figure 3.**
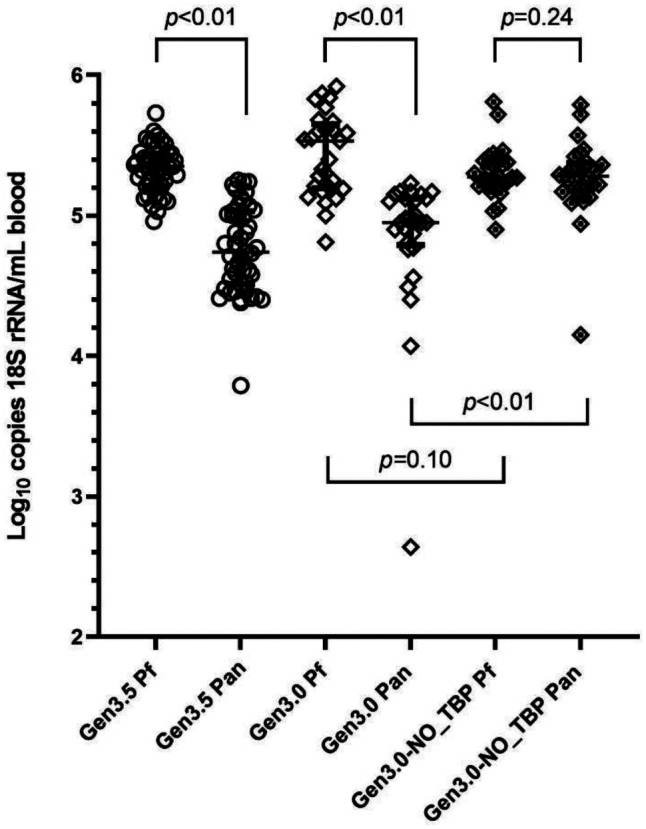
Distribution and comparisons of *Plasmodium falciparum* 18S rRNA copy numbers in ring-stage parasites by different assay versions. *Plasmodium falciparum* 18S rRNA copies per mL blood samples were reported by *P. falciparum* qRT-PCR (Pf) and pan *Plasmodium* qRT-PCR (Pan) for the Gen3, the Gen3.5, and the Gen3-NO-TBD assays. Median, 25% percentile, and 75% percentiles are marked. *P* values are shown.

Quantitative reverse transcription PCR of the *P. falciparum* and pan-*Plasmodium* targets by the Gen3.0 and Gen3.5 assays provided quantitative estimates of the 18S rRNA content of individual ring-stage *P. falciparum* parasites. Comparison of qRT-PCR results for these samples showed that the pan-*Plasmodium* target-based estimates of both assays were lower than *P. falciparum* target-based estimates (Figure 3). For these samples, the difference between median values generated by *P. falciparum* and pan-*Plasmodium* qRT-PCR in the Gen3.0 assay was 0.53 log_10_ copies/parasite (Wilcoxon test *P* < 0.01) and the difference between medians in the Gen3.5 assay was 0.56 log_10_ copies/parasite blood (*P* < 0.01).

**Figure 4.**
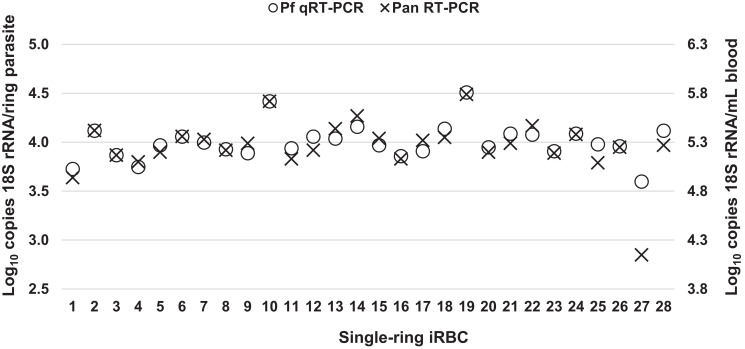
Copy number quantification for samples containing one iRBC per sample. The copy numbers of *Plasmodium* 18S rRNA are shown for each of 28 individually assayed samples containing a single ring per sample. Testing was performed using multiplex *P. falciparum* (Pf, open circles) qRT-PCR and Pan *Plasmodium* (Pan, crosses) qRT-PCR of the Gen3.0-NO_TBP assay. The left y-axis displays the values when calculated on a per-parasite basis and the right y-axis displays the data when multiplied to reflect copies per mL of whole blood.

Based on recent experience,^12^ we hypothesized that quantitative differences in the ultra-low density range may be due to competition between the Pan *Plasmodium* and host TBP RT-PCR. To investigate, the Gen3.0 qRT-PCR assay was performed with and without the host TBP RT-PCR reagents (Gen3-NO_TBP) on 28 samples each containing a single-ring iRBC. Pan-*Plasmodium* estimates generated without the TBP reagents matched *P. falciparum* estimates (*P* = 0.24 shown in Figure 3); the difference between each paired result was < 0.2 log_10_ copies/mL blood (Figure 4). Host TBP RT-PCR significantly shifted the pan-*Plasmodium* estimates (difference of two medians = 0.33; Mann-Whitney test *P* < 0.01 versus Gen3.0 pan-*Plasmodium* shown in Figure 3). While the TBP also shifted *P. falciparum* estimates, the magnitude was minimal (difference of two medians = −0.26; *P* = 0.10 versus Gen3.0 *P. falciparum* shown in Figure 3). Pan-*Plasmodium* qRT-PCR in the triplex settings of the Gen3.0 and Gen3.5 assays revealed lower sensitivities compared with those for *P. falciparum* qRT-PCR (Figure 3) when testing single-ring iRBCs in this study. Many factors may affect the efficiencies of singleplex and multiplex RT-PCRs, such as guanine–cytosine content, the length of primers, and the secondary structures in targeted regions of the 18S rRNA. At higher densities, differences of medians were < 0.2 log_10_ copies/mL for Gen3.0 and Gen3.5 shown in Supplemental Figure 3A and B, respectively. These observations suggested that competition with TBP is not a factor when *Plasmodium* 18S rRNA is abundant.

Without TBP RT-PCR, *P. falciparum* and pan *Plasmodium* qRT-PCRs yielded very similar measures of 18S rRNA for each single-ring parasite (Figure 4). The median values for both *P. falciparum* and pan-*Plasmodium* qRT-PCR were 3.98 log_10_ copies/parasite (*P. falciparum* estimation range, 3.60–4.51 log_10_ copies/parasite; interquartile 3.91–4.09 log_10_ copies/parasite) (Table 2). These 18S rRNA content measures were similar to those estimated earlier using hand-diluted standard curves.^1^^,^^9^

**Table 2:** Range of 18S rRNA content per parasite (log_10_ copies 18S rRNA per ring-stage parasite)

	Gen3.0		Gen3.5		Gen3.0_NO-TBP	
	*P. falciparum*	*Pan-Plasmodium*	*P. falciparum*	*Pan-Plasmodium*	*P. falciparum*	*Pan-Plasmodium*
Minimum	3.51	1.34	3.66	2.49	3.60	2.85
25% Percentile	3.89	3.49	3.91	3.21	3.91	3.88
Median	4.23	3.65	4.05	3.44	3.98	3.98
75% Percentile	4.35	3.82	4.14	3.73	4.09	4.08
Maximum	4.62	3.93	4.43	3.95	4.51	4.49

Given this information, the *P. falciparum* qRT-PCR of the triplex assays provided the most accurate estimate of per-parasite copy number (Table 2). The median copy number by the Gen3.5 *P. falciparum* qRT-PCR was 4.05 log_10_ copies 18S rRNA per single-ring iRBC (range 3.66–4.43 log_10_ copies/parasite; interquartile range 3.91–4.14 log_10_ copies/parasite), in agreement with those derived from the NO-TBP assay estimates (Mann–Whitney *t* test *P* = 0.39). Thus, these data support using the Gen3.5 assay *P. falciparum* qRT-PCR channel for primary quantification in future studies.

## DISCUSSION

This study adopted a published procedure to stain DNA and RNA of live *P. falciparum* culture and visualize iRBCs by IFC.^11^ Bright-field images revealed single ring stage parasites in the iRBC gated with the lowest DNA and RNA content. HO (DNA) and TO (RNA)-stained single dots were co-localized with each ring in each iRBC, confirming the presence of a parasite nucleus and abundant RNA within each ring. Some cells presented with higher intensities of HO- and TO-staining florescence showed two to three visible rings per RBC. Other iRBCs with even higher intensities of HO- and TO-staining florescence demonstrated structures that correspond to trophozoites and/or schizonts. Staining with two dyes thus allowed for adequate separation of single-ring iRBCs from uRBCs, as well as discrimination of iRBCs containing multiple rings, trophozoites, and/or schizonts, consistent with the original publication. Here, with this knowledge, the same gating was used to sort single-ring iRBCs into individual wells of 96-well plates for subsequent testing by 18S rRNA qRT-PCR. Among single-ring iRBCs, 18S rRNAs were reliably detected in > 95% of cells by all qRT-PCR assays. None of the sorted uRBCs were positive for any *Plasmodium* 18S rRNA, confirming the specificity of the assay and the sorting approach. This work demonstrates that FACS can be used to overcome limiting sampling described by the Poisson distribution and confirmed the ability of the UW qRT-PCR assays to detect a single ring-stage parasite in 50 µL of whole blood. Although the 18S rRNA per-parasite content was previously estimated using hand-pipetted bulk parasite dilutions,^1^^,^^6^^,^^8^^,^^9^ this study is the first to report on definitive individual parasites and the 18S rRNA content of 3.5–4.6 log_10_ copies/parasite agrees with previous estimates.

Thus, this study confirms the exquisite analytical sensitivity of the UW qRT-PCR assays at one parasite per 50 µL of whole blood (equivalent to 20 parasites/mL of blood). Thus, the main driver of analytical sensitivity for this approach is the volume of blood subjected to nucleic acid extraction. The standard volume is 0.05 mL of blood, which is diluted in 2 mL lysis buffer and extracted. However, we previously showed that if the blood volume was scaled up 10-fold (0.5 mL), then the analytical sensitivity could be improved 10-fold (two parasites/mL blood).^8^ This is possible because of the high 18S rRNA content per parasite, such that even with dilution of 0.5 mL of blood, hundreds of 18S rRNA molecules are nonetheless propagated through the extraction and into the qRT-PCR.^8^

The probabilities of having a parasite present in a 50 µL blood sample are described by the Poisson distribution. For example, at a nominal density of 20, 40, 60, or 80 parasites/mL, the probabilities would be 63.2%, 86.5%, 95.0%, and 98.2%, respectively. The FACS-based approach allows us to confirm that a positive result at 20 parasites/mL is definitively parasite-derived and does not represent background signal. If we were to use classical methods to verify the analytical sensitivity, we would have been potentially forced to disregard such results as “negative” because of sampling statistics. Therefore, for some patients at a nominal density of 20 parasites/mL, the sample will in fact appear negative because a parasite was not contained in the test aliquot. However, when a qRT-PCR signal is detected at this level, our data showed that this is a reliable, reproducible, and parasite-derived true positive.

There are benefits to reporting positivity for such low parasite densities. First, these initial positives in CHMI studies denote when parasites are first released from the liver, which helps to assess the performance of experimental vaccines and drugs. Second, participants in CHMI experience none or mild malaria-related symptoms, because this qRT-PCR positivity precedes symptoms and blood smear positivity by several days.^6^ Third, outside of CHMI trials, added sensitivity allows for improved understanding of the prevalence and density distribution of infections in a community or a study. A general agreement in infectious diseases is to use the most sensitive test to rule out a particular disease or infection, and in this regard, highly sensitive molecular tests can provide the greatest amount of epidemiological data. For example, very low positive molecular diagnostic results after consecutive negative results could suggest recrudescence or relapse of *Plasmodium* infection.^13^^–^^15^ Similarly, some such results could be due to gametocytemia with or without the presence of asexual parasites, which could inform studies of transmission and the infectious reservoir.^16^^–^^19^ Thus, reporting positivity for low-density *Plasmodium* infection can help improve the diagnosis of asymptomatic and/or chronic infections, which is critical to achieving a long-standing goal of malaria elimination and eradication.^20^^,^^21^

In the UW qRT-PCR assay, commercially sourced Pf-Armored RNA serves as an absolute calibration standard to accurately measure *P. falciparum* 18S rRNA in blood samples.^6^ Armored RNA technology has been widely used to make calibration standards for FDA-approved RNA-targeted molecular quantitative assays.^22^^–^^25^ This approach was uniquely wedded to our parasite where each parasite contains an extremely large number of molecular diagnostic target sequences. In contrast, the digital PCR (dPCR) concept has been adapted to droplet- and microchip-based technologies to partition, amplify, and determine absolute copy numbers with no need for a calibration curve.^26^ Within the dynamic range (i.e., across 3–4 logs), dPCR performs better than qPCR in terms of accuracy and precision,^27^^,^^28^ however, dPCR methodology is used less often for molecular diagnostics because of reagent cost and device availability.^26^^,^^29^ At this time, reverse-transcription dPCR (RT-dPCR) is even less common than dPCR because additional reverse transcription steps are required, which can introduce varied reaction efficiencies.^26^^,^^30^ Despite these challenges, some laboratories are using dPCR and RT-dPCR to estimate *Plasmodium* genomic DNA and gametocyte mRNA markers in field samples.^29^ An estimated 3.5–4.6 log_10_ 18S rRNA per single ring-stage iRBC indicates that the 18S rRNA target is much more abundant than the RT-dPCR dynamic range is meant to accommodate. Thus, qRT-PCR calibration using Pf-Armored RNA is the appropriate method for absolute calibration.

In conclusion, this study demonstrated that FACS sorted single-ring iRBCs were reliably detected over 95% of the time using multiple generations of the UW qRT-PCR assay. This confirmed the reported analytic sensitivity of this approach as 20 parasites/mL, which is primarily defined by sampling volume. The median copy number of 18S rRNA per ring was 9,550 copies, consistent with our previous albeit less direct estimations. With this added validation, results from 18S rRNA-based qRT-PCR assays can be relied upon to qualitatively and quantitatively inform efforts to combat malaria even for extremely low-density samples.

## Supplemental Material


Supplemental materials
