# Supplementary material for: Flow Cytometric Sorting of Infected Erythrocytes Demonstrates Reliable Detection of Individual Ring-Stage Plasmodium falciparum Parasites by Plasmodium 18S rRNA Reverse Transcription Polymerase Chain Reaction
Source: Am J Trop Med Hyg. 2022 Apr 11;106(6):1653–9. doi: 10.4269/ajtmh.21-1226 (PMC9209910; doi:10.4269/ajtmh.21-1226)

Supplemental Figure S1. Microscopic blood smear of *P. falciparum* cultures. Examples of iRBCs containing (**A**) a single ring, (**B**) multiple rings, (**C**) early trophozoites, and (**D**) late trophozoites.

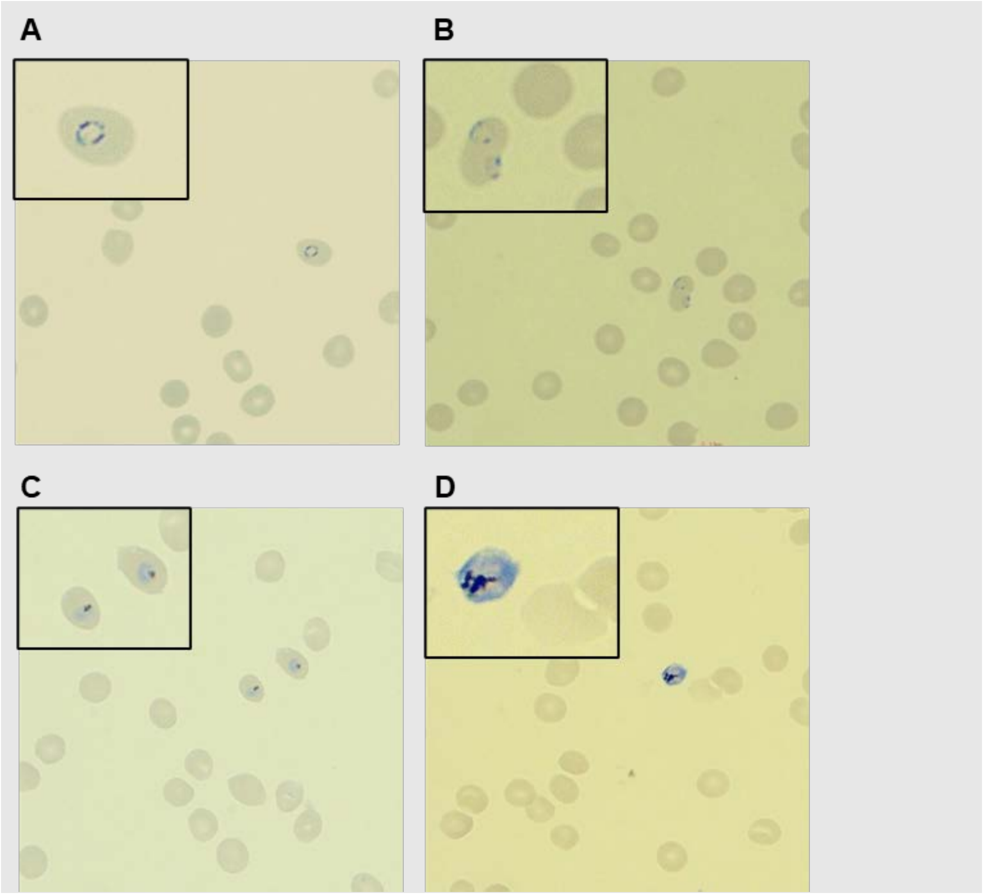

Supplemental Figure S2. Flow sort single-ring iRBC of *P. falciparum* culture. Distribution of uninfected RBCs and iRBCs in synchronous (**A**) and asynchronous (**B**) *P. falciparum* cultures stained by Hoechst 33342 and thiazole orange, which emitted fluorescence filtered by DAPI and FITC, respectively. Infected RBCs and single-ring iRBCs were gated and marked with "++" and Rings, respectively.

A. **B.**

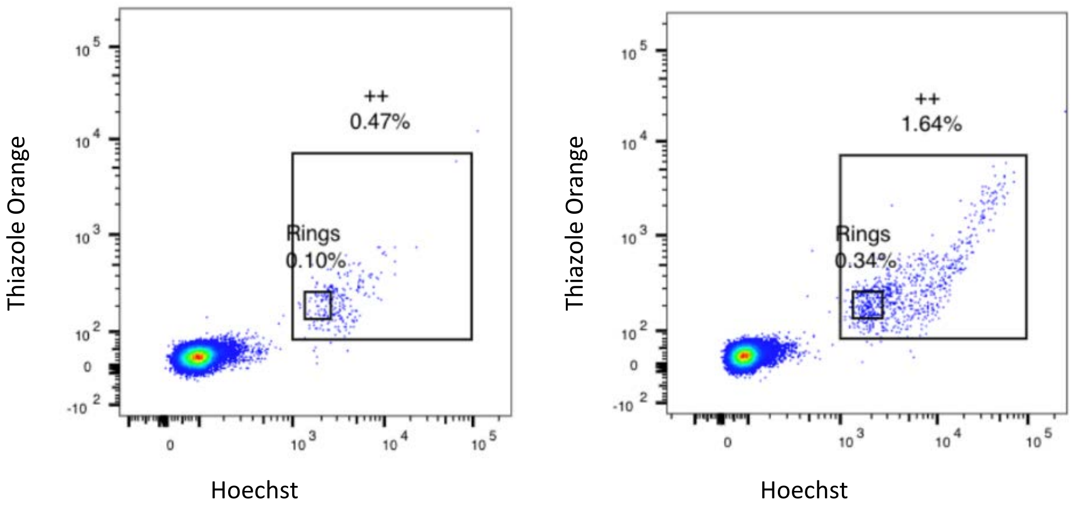

Supplemental Figure S3. Subpatent parasitemia measured by the Gen3.0 (A) and Gen3.5 (B) assays. Synchronous *P. falciparum* cultures were used to prepare High Pos, Mid Pos and Low Pos controls of different densities. Nominal densities of them were 4×10^5, 2×10^3, 1.0×10^2 parasites/mL blood, respectively, used for evaluation of the Gen3.0 assay and they were 9×10^5, 4×10^3, 2×10^2 parasites/mL blood, respectively, for the Gen3.5 assay. Multiple replicates were run by *P. falciparum* qRT-PCR (Pf) and pan *Plasmodium* qRT-PCR (Pan) of each assay.

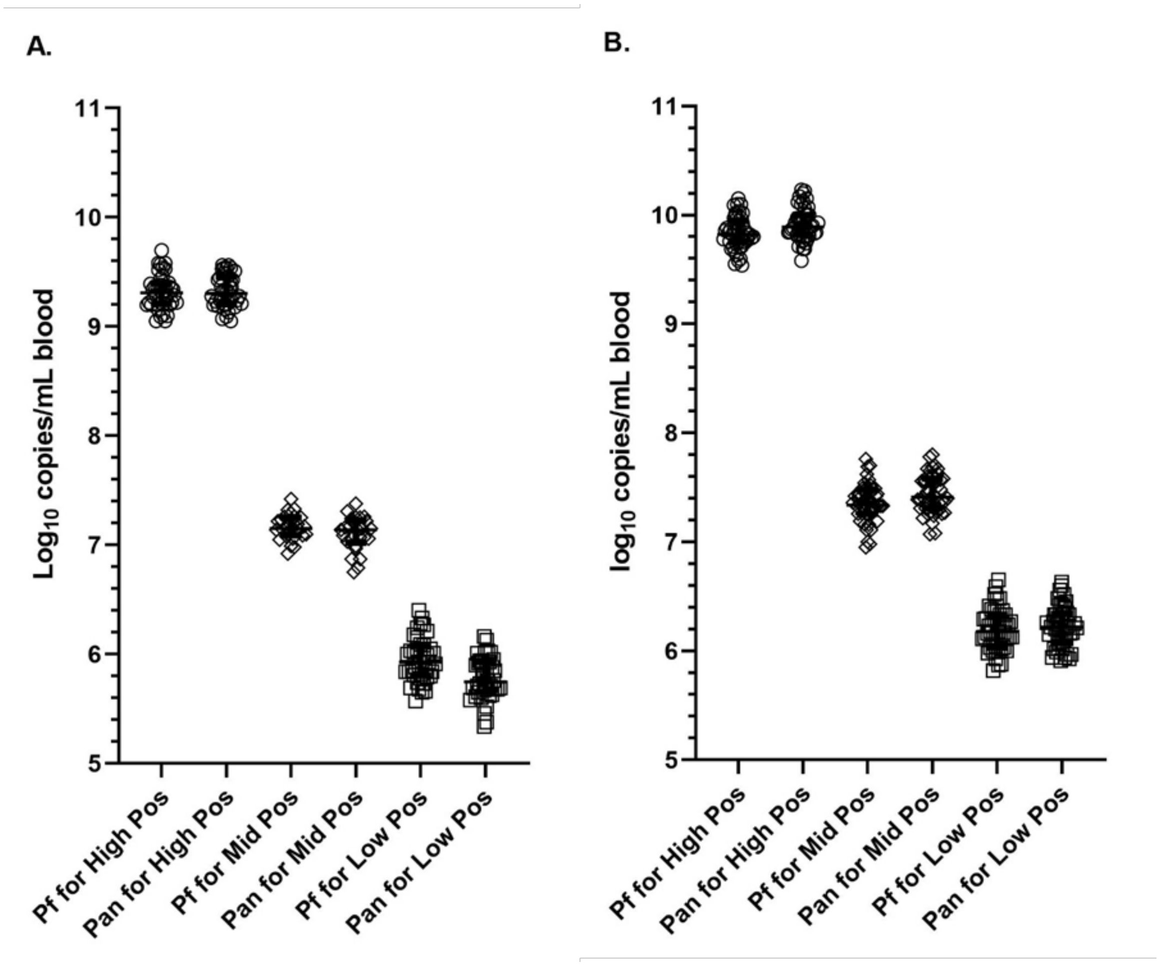

Supplement: Supplementary file 1 [file tpmd211226.SD1.pdf]
